# The Danger of Chloroform Anesthesia by Gaslight

**Published:** 1898-10

**Authors:** Eugene R. Corson

**Affiliations:** Savannah, Ga.


### THE DANGER OF CHLOROFORM ANESTHESIA BY GASLIGHT.

BY EUGENE R. CORSON, M.D.,

SAVANNAH, GA.

The danger of chloroform anesthesia by gaslight has been again brought to the notice of the profession by a death in the Catholic hospital at Herne, in Westphalia. A case of gunshot wound in the abdomen was operated upon at night under gaslight, and the decomposition of the chloroform vapor suffocated two surgeons and several nurses, one of the latter dying from the effects of it. How the poor patient fared is not known; but think of the danger to the patient under such circumstances!

Last year, in our own city, a case of appendicitis operated upon by gaslight showed the danger of the use of chloroform in the presence of a flame which could oxidize and decompose the chloroform vapor. The suffocating fumes of the products formed almost drove the occupants out of the room.

I can find nowhere any statement of the exact reaction which takes place, but I have worked out the following formulæ, which I believe represent the correct reactions. I have submitted them to a good chemist and he has pronounced them correct. According to the completeness of the combustion or oxidation, two reactions are possible. If the oxidation is complete we have the following reaction:

$$4\ CHCl_3 + 5\ O_2 = 2\ H_2O + 4\ CO_2 + 6\ Cl_2.$$

Here the really poisonous product formed is the free chlorine, a most dangerous gas when existing to any extent in the atmosphere.

When the oxidation is not so complete, we have a quite different reaction, with the formation of formaldehyde in addition to the chlorine. The reaction may be thus represented:

$4\ CHCl_3 + 3\ O_2 = 2\ CH_2O + 2\ CO_2 + 6\ Cl_2$, or, four molecules of chloroform with three molecules of oxygen produce two molecules of formaldehyde, two molecules of carbon dioxide, and six molecules of chlorine gas. It is at once apparent how deadly such a decomposition must be in an ordinary room with ordinary ventilation, and a number of cases on record bear witness to it. It is evident that illuminating gas in itself is not the trouble, but it is the flame, which, burning in an atmosphere charged with chloroform vapor, causes its combustion and decomposition. Any other flame would do the same—as an alcohol flame or a flame from a kerosene lamp, for example.

It may be interesting to note the reactions from combustion of some allied compounds. Methyl alcohol, for example, under the active combustion which takes place in the ordinary spirit lamp, is represented as follows:

$2\ CH_3HO + 3\ O_2 = 2\ CO_2 + 4\ H_2O$. In other words, it is decomposed into carbon dioxide and water.

Note again, where the oxidation of the methyl alcohol is not so complete we have another reaction, as follows:

$CH_3HO + O_2 = HC\ HO_2 + H_2O$, or, one molecule of methyl alcohol and one molecule of oxygen produce one molecule of formic acid and one molecule of water.

Reduce still further the amount of oxygen, as seen when the methyl alcohol is burnt in a lamp constructed for the purpose, and we have the production of formaldehyde, so largely employed today as a disinfectant and germicide:

$2\ CH_3HO + O_2 = 2\ CH_2O + 2\ H_2O$, $CH_2O$ representing the formaldehyde.

The inflammability of sulphuric ether is more generally recognized, but the ether must be brought directly in contact with the flame to explode. I once witnessed a very serious accident of this kind, where a surgeon and nurse were very severely burnt and a number of instruments destroyed. The products of such combustion, however, are not so bad; witness the reaction thus chemically stated:

$C_4H_{10}O + 6\ O_2 = 5\ H_2O + 4\ CO_2$.

Ether vapor in the atmosphere coming in contact with a flame would therefore not be serious under the conditions which hold ordinarily, and ether anesthesia could be used by gaslight.

Electric lighting is so generally superseding gas that there is less opportunity for ignorance to cause trouble, and such an accident as recently occurred will be very rare.

